# Cost-effectiveness analysis of toripalimab plus chemotherapy versus standard chemotherapy in first-line treatment for extensive-stage small cell lung cancer: perspectives from the United States and China

**DOI:** 10.3389/fphar.2025.1616942

**Published:** 2025-08-20

**Authors:** Ming Ouyang, Jiangbo Wang, Gaofeng Zhang, Bei Huang, Lin Deng, Lian Deng, Wenwang Lang

**Affiliations:** ^1^ Department of Pharmacy, Nanxishan Hospital of Guangxi Zhuang Autonomous Region, Guilin, China; ^2^ Department of Oncology, Nanxishan Hospital of Guangxi Zhuang Autonomous Region, Guilin, China

**Keywords:** cost-effectiveness, extensive-stage small-cell lung cancer, toripalimab, chemotherapy, Markov model

## Abstract

**Background:**

Toripalimab combined with chemotherapy has demonstrated significant clinical advantages in improving overall survival compared with chemotherapy alone as a first-line treatment for extensive-stage small-cell lung cancer (ES-SCLC).

**Method:**

An economic evaluation was conducted using a Markov state-transition model to reflect the perspectives of the United States payer and Chinese healthcare systems. Primary outcomes included quality-adjusted life-years (QALYs), incremental cost-effectiveness ratio (ICER), incremental net health benefit (INHB), and incremental net monetary benefit (INMB).

**Results:**

Base-case analysis indicated that incorporating toripalimab into chemotherapy produced an ICER of $45,629.27 per QALY, exceeding China’s willingness-to-pay (WTP) threshold of $38,042.49 per QALY. Subgroup analyses revealed ICERs of $22,345.99 and $30,867.38 per QALY for patients with low intratumor heterogeneity (ITH-L) and A11+/B62- histology, respectively, both below the China WTP threshold. In contrast, in the United States, the additional cost led to unfavorable ICERs of $842,855.23, $328,694.61, and $520,412.03 per QALY for the overall population, the ITH-L subgroup, and the A11+/B62− subgroup, respectively, each exceeding the United States WTP threshold of $150,000.00.

**Conclusion:**

The combination of toripalimab and chemotherapy was not found to be a cost-effective first-line treatment for ES-SCLC in China or the United States, except for patients in China with ITH-L and A11+/B62- histology.

## Introduction

Lung cancer remains a leading cause of cancer-related mortality globally and is the second most common cancer diagnosed (Rudin et al., 2021; Sung et al., 2021; Oronsky et al., 2022), with small cell lung cancer (SCLC) accounting for approximately 15% of cases. Extensive-stage SCLC (ES-SCLC), comprising 80%–85% of SCLC diagnoses, is associated with poor prognosis and limited survival rates despite advances in therapy. Epidemiologically, China reports approximately 150,000 new ES-SCLC cases annually, while the United States sees around 40,000 cases, with most patients presenting at an advanced stage at diagnosis (Sung et al., 2021). Traditional first-line treatment, consisting of platinum-based chemotherapy combinations, has historically offered median survival rates of only 10 months and 5-year survival rates below 5% (Rossi et al., 2012; Rudin et al., 2016; Sathiyapalan et al., 2022).

The emergence of immune checkpoint inhibitors (ICIs) has revolutionized the treatment paradigm for ES-SCLC. Several phase III trials have demonstrated better survival with ICIs, such as atezolizumab, durvalumab, adebrelimab, and serplulimab combined with chemotherapy (Horn et al., 2018; Paz-Ares et al., 2019; Cheng et al., 2022; Wang et al., 2022; Cheng et al., 2024a). Toripalimab, a novel PD-1 antibody with unique binding properties (Rajasekaran et al., 2024), showed enhanced efficacy in combination with chemotherapy in the EXTENTORCH trial. This study established the benefits of toripalimab in improving progression-free survival (PFS) by 5.8 months and overall survival (OS) by 14.6 months in ES-SCLC patients, as observed in the EXTENTORCH trial (Cheng et al., 2024b).

Although the clinical efficacy of toripalimab is apparent, its cost-effectiveness remains unclear. High drug costs pose significant financial challenges, particularly for patients in middle-income countries such as China, where healthcare coverage may be limited and out-of-pocket expenses can represent a substantial burden. For example, the high price of toripalimab can exceed the annual income of many families, requiring policy interventions or subsidy programs to ensure affordability. Additionally, toripalimab has entered the United States market and may soon be included in NCCN guidelines, underscoring the need for cost-effectiveness data to inform clinical and policy decisions in both regions. This study evaluated the cost-effectiveness of toripalimab plus chemotherapy versus chemotherapy alone for ES-SCLC treatment from the perspectives of Chinese and United States healthcare, focusing on the general population and subgroups characterized by low intratumor heterogeneity (ITH-L), as defined by a Mutant-Allele Tumor Heterogeneity score of less than 29, and HLA-A11+/B62- haplotypes. In the EXTENTORCH study, patients with ITH-L in the toripalimab group experienced significantly improved PFS and OS. Moreover, patients with the HLA-A11+ HLA-B62− haplotype exhibited extended clinical benefits following toripalimab treatment.

## Methods

### Patients and intervention

This study was reported according to the Consolidated Health Economic Evaluation Reporting Standards (CHEERS) checklist (Husereau et al., 2022). The targeted patients were ≥18 years of age and had histologically or cytologically confirmed ES-SCLC. Other key characteristics aligned with the EXTENTORCH study.

The induction phase comprised four 21-day cycles of intravenous (IV) toripalimab 240 mg or placebo administered every 3 weeks (Q3W), combined with etoposide (100 mg/m^2^ intravenously on days 1–3 of each cycle) and carboplatin (area under the plasma or serum concentration-time curve = 5) on the first day of each cycle. Subsequently, maintenance therapy with 240 mg IV toripalimab or placebo Q3W continued until disease progression, loss of clinical benefit, unacceptable toxicity, or withdrawal of consent. Tumor imaging evaluations were conducted 6 weeks after initiating treatment and subsequently every 6 weeks for the first 54 weeks, then every 12 weeks until disease progression, loss of follow-up, death, withdrawal of consent, or initiation of new anticancer therapy.

Among the patients, 55.2% in the toripalimab group and 69.4% in the placebo group received additional systemic anticancer treatments after discontinuing the study medications. Common treatments in the toripalimab group included conventional chemotherapies (49.3%), tyrosine kinase inhibitors (32.3%), and PD-1/PD-L1 inhibitors (13.9%), while in the placebo group, these were 59.4%, 43.8%, and 25.1%, respectively.

As the median progression-free survival (mPFS) did not exceed 6 months in either group, the selected chemotherapies adhered to the National Comprehensive Cancer Network (NCCN) (NCCN, 2025), the Chinese Society of Clinical Oncology (CSCO) guidelines (Yaoch, 2025), and the EXTENTORCH trial. The recommended combinations included topotecan with cisplatin, anlotinib as a tyrosine kinase inhibitor, and toripalimab as a PD-1/PD-L1 inhibitor. Body surface area and creatinine clearance rates were assumed to be similar to those reported in previous studies.

The cost implications of adverse events (AEs) were evaluated using data from the RATIONALE-312 trial. The focus was exclusively on grade 3 or 4 serious adverse events (SAEs), with an incidence rate above 5%. These SAEs included anemia, reduced platelet and neutrophil counts, hyponatremia, hypokalemia, and pneumonia (Tables 1–3).

**TABLE 1:** Model parameters (China).

Parameters		Baseline value	Range		Distribution	Reference
			Minimum	Maximum		
Survival model for OS						
Toripalimab plus chemotherapy		Shape = 2.558 Scale = 17.166			Loglogistic	Cheng et al. (2024b)
Placebo plus chemotherapy		Shape = 2.734 Scale = 15.127			Loglogistic	Cheng et al. (2024b)
Survival model for PFS						
Toripalimab plus chemotherapy		Shape = 1.839 Scale = 22.624			Loglogistic	Cheng et al. (2024b)
Placebo plus chemotherapy		Shape = 2.603 Scale = 15.128			Loglogistic	Cheng et al. (2024b)
Survival model for OS (ITH-L)						
Toripalimab plus chemotherapy		Shape = 1.839 Scale = 22.624			Loglogistic	Cheng et al. (2024b)
Placebo plus chemotherapy		mu = 2.4562 sigma = 0.6261 Q = −0.9901			Gengamma	Cheng et al. (2024b)
Survival model for PFS (ITH-L)						
Toripalimab plus chemotherapy		Meanlog = 2.169 Sdlog = 0.876			Lognormal	Cheng et al. (2024b)
Placebo plus chemotherapy		Shape = 3.589 Scale = 6.302			Loglogistic	Cheng et al. (2024b)
Survival model for OS(A11+/B62-)						
Toripalimab plus chemotherapy		Meanlog = 3.062 Sdlog = 0.507			Lognormal	Cheng et al. (2024b)
Placebo plus chemotherapy		Meanlog = 2.733 Sdlog = 0.638			Lognormal	Cheng et al. (2024b)
Survival model for PFS (A11+/B62-)						
Toripalimab plus chemotherapy		Meanlog = 2.154 Sdlog = 0.643			Lognormal	Cheng et al. (2024b)
Placebo plus chemotherapy		Shape = 3.078 Scale = 5.934			Loglogistic	Cheng et al. (2024b)
Drug cost, $/per cycle						
Cost of toripalimab		346.07	276.86	415.28	Gamma	Yaoch (2025)
Cost of carboplatin		51.26	41.01	61.51	Gamma	Yaoch (2025)
Cost of cisplatin		35.03	28.02	42.04	Gamma	Yaoch (2025)
Cost of etoposide		6.63	5.30	7.96	Gamma	Yaoch (2025)
Cost of topotecan		246.69	197.35	296.03	Gamma	Yaoch (2025)
Cost of anlotinib		563.24	450.59	675.89	Gamma	Yaoch (2025)
Cost of the laboratory test		92.99	74.39	111.59	Gamma	Zhu et al. (2022)
Enhanced CT/MRI		241.67	193.34	290.00	Gamma	Yaoch (2025)
Cost of end-of-life		1,460.30	1,168.24	1752.36	Gamma	Cao et al. (2022), Liu et al. (2023a)
Best supportive care		345.60	276.48	414.72	Gamma	(17)
Cost of drug administration per unit	Preventive medication per intravenous administration	93.93	75.14	112.72	Gamma	Cao et al. (2022), Liu et al. (2023a)
	Infusion fee per intravenous administration	1.86	1.49	2.23	Gamma	Cao et al. (2022), Liu et al. (2023a)
	Preventive medication	39.14	31.31	46.97	Gamma	Cao et al. (2022), Liu et al. (2023a)
Proportion of receiving subsequent treatment						
Toripalimab plus chemotherapy group		55.20%	44.16%	66.24%	Beta	Cheng et al. (2024b)
Placebo plus chemotherapy		69.40%	55.52%	83.28%	Beta	Cheng et al. (2024b)
Subsequent treatment in Toripalimab plus Chemotherapy group						
Cytotoxic agents		49.30%	39.44%	59.16%	Beta	Cheng et al. (2024b)
Tyrosine kinase inhibitors		32.30%	25.84%	38.76%	Beta	Cheng et al. (2024b)
PD-1/PD-L1 inhibitors		13.90%	11.12%	16.68%	Beta	Cheng et al. (2024b)
Subsequent treatment in Placebo plus Chemotherapy group						
Cytotoxic agents		59.40%	47.52%	71.28%	Beta	Cheng et al. (2024b)
Tyrosine kinase inhibitors		43.80%	35.04%	52.56%	Beta	Cheng et al. (2024b)
PD-1/PD-L1 inhibitors		25.10%	20.08%	30.12%	Beta	Cheng et al. (2024b)
Discount rate (China)		5%	4.00%	6.00%	Beta	
BMI/m2		1.72				
Weight/kg		65.00				
$1 = ¥7.0467		38,042.49				

ITH-L, low intratumor heterogeneity; OS, overall survival; PFS, progression-free survival; PD, progression disease; BMI, body mass index.

**TABLE 2:** Model parameters of AEs(China).

Parameters	Baseline value	Range		Distribution	Reference
		Minimum	Maximum		
*Cost of AEs, $*					
Anaemia	138.75	112.32	166.50	Gamma	Zhu et al. (2022)
Decreased platelet count	1,505.92	1,219.06	1807.10	Gamma	Zhu et al. (2022)
Decreased neutrophil count	115.01	80.92	138.01	Gamma	Zhu et al. (2022)
Hyponatraemia	3,223.00	2,578.40	3,867.60	Gamma	Liu et al. (2023b)
Hypokalaemia	3,000.00	2,400.00	3,600.00	Gamma	Liu et al. (2023b)
Pneumonia	2,105.00	1,684.00	2,526.00	Gamma	Jiang and Wang (2022)
Utilities					
Utility of PFS	0.69	0.55	0.83	Beta	Vedadi et al. (2021)
Utility of PD	0.60	0.48	0.72	Beta	Vedadi et al. (2021)
Disutility estimates					
Anemia	0.07	0.06	0.09	Beta	Zhu et al. (2022)
Decreased platelet count	0.05	0.04	0.06	Beta	Zhu et al. (2022)
Decreased neutrophil count	0.20	0.16	0.24	Beta	Zhu et al. (2022)
Hyponatraemia	0.04	0.03	0.05	Beta	Liu et al. (2023b)
Hypokalaemia	0.04	0.03	0.05	Beta	Liu et al. (2023b)
Pneumonia	0.09	0.07	0.11	Beta	Zhu et al. (2021)
Risk for main AEs in Toripalimab plus Chemotherapy group					
Anemia	30.60%	24.48%	36.72%	Beta	Cheng et al. (2024b)
Decreased platelet count	24.80%	19.84%	29.76%	Beta	Cheng et al. (2024b)
Decreased neutrophil count	74.30%	59.44%	89.16%	Beta	Cheng et al. (2024b)
Hyponatraemia	6.30%	5.04%	7.56%	Beta	Cheng et al. (2024b)
Hypokalaemia	5.90%	4.72%	7.08%	Beta	Cheng et al. (2024b)
Pneumonia	6.80%	5.44%	8.16%	Beta	Cheng et al. (2024b)
Risk for main AEs in Placebo plus Chemotherapy group					
Anemia	34.70%	27.76%	41.64%	Beta	Cheng et al. (2024b)
Decreased platelet count	34.30%	27.44%	41.16%	Beta	Cheng et al. (2024b)
Decreased neutrophil count	75.00%	60.00%	90.00%	Beta	Cheng et al. (2024b)
Hyponatraemia	6.50%	5.20%	7.80%	Beta	Cheng et al. (2024b)
Hypokalaemia	6.50%	5.20%	7.80%	Beta	Cheng et al. (2024b)
Pneumonia	3.20%	2.56%	3.84%	Beta	Cheng et al. (2024b)

PFS, progression-free survival; PD, progression disease; AE, adverse event.

**TABLE 3:** Model parameters (United States).

Parameters	Baseline value	Range		Distribution	Reference
		Minimum	Maximum		
Drug cost, $/per cycle					
Cost of Toripalimab	8,892.03	7,113.62	10,670.44	Gamma	Centers_for_Medicare_and_Medicaid_Services (2025)
Cost of Carboplatin	55.83	44.66	67.00	Gamma	Centers_for_Medicare_and_Medicaid_Services (2025)
Cost of Cisplatin	45.79	36.63	54.95	Gamma	Centers_for_Medicare_and_Medicaid_Services (2025)
Cost of Etoposide	62.76	50.21	75.31	Gamma	Centers_for_Medicare_and_Medicaid_Services (2025)
Cost of Topotecan	2,720.26	2,176.21	3,264.31	Gamma	Centers_for_Medicare_and_Medicaid_Services (2025)
Cost of the laboratory test	111.65	89.32	133.98	Gamma	Centers_for_Medicare_and_Medicaid_Services (2025)
Enhanced CT/MRI	438.21	350.568	525.85	Gamma	Centers_for_Medicare_and_Medicaid_Services (2025)
Cost of end-of-life	21,603.00	17,282.40	25,923.60	Gamma	Shao et al. (2023)
Best supportive care	1,447.79	1,158.23	1737.35	Gamma	Shao et al. (2023)
Cost of drug administration first hour	142.55	114.04	171.06	Gamma	Liu et al. (2021)
Administration intravenous, additional hour	30.68	24.54	36.82	Gamma	Liu et al. (2021)
Cost of AEs, $					
Anaemia	7,941.00	6,352.80	9,529.20	Gamma	Shao et al. (2023)
Decreased platelet count	13,105.00	10,484.00	15,726.00	Gamma	Shao et al. (2023)
Decreased neutrophil count	13,105.00	10,484.00	15,726.00	Gamma	Shao et al. (2023)
Hyponatraemia	4,685.00	3,748.00	5,622.00	Gamma	Wong et al. (2018)
Hypokalaemia	4,685.00	3,748.00	5,622.00	Gamma	Wong et al. (2018)
Pneumonia	10,756.00	8,604.80	12,907.20	Gamma	Wong et al. (2018)
Discount rate	3%	4.00%	6.00%	Beta	
BMI/m2	1.79				
Weight/kg	65				

AE, adverse event, BMI, body mass index.

### Model structure

**FIGURE 1:**
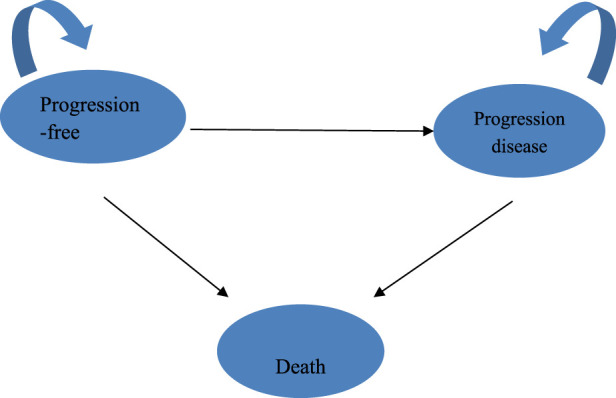
Markov model structure.

A three-state Markov model (“progression-free survival,” “progressive disease,” and “death”) was used to simulate treatment outcomes over a 10-year horizon (Figure 1). The model outcomes were developed and analyzed using the TreeAge Pro 2022 software (Williamstown, MA, United States) and R software (version 4.2.3, Vienna, Austria). The model inputs included the survival curves for PFS and OS. All patients entered the model in the PFS state and received chemotherapy alone or chemotherapy combined with toripalimab until disease progression or unacceptable toxicity was observed. Patients transitioning to the PD state received subsequent therapies following the discontinuation of toripalimab or placebo in combination with chemotherapy. The proportion of patients in the PD state was determined using the area under the OS curve, proportion of patients alive with OS, proportion of patients alive with PFS, and difference between the OS and PFS curves. Cost-effectiveness analyses (CEAs) were conducted from the perspective of the United States payer and Chinese healthcare system. Only direct medical costs were included in the United States perspective, whereas the Chinese perspective considered broader healthcare system costs (Dieleman et al., 2020).

### Outcomes

The outcomes were measured in quality-adjusted life years (QALYs) and costs in United States dollars. Both costs and utilities were discounted annually at 3% in the United States and 5% in China (Su et al., 2021; Yue et al., 2021). In China, costs were updated to 2023 values using the local consumer price index and converted to United States dollars based on an exchange rate of $1 = ¥7.0467. CEAs were performed, with the results expressed as incremental cost-effectiveness ratios (ICERs). ICERs were calculated as the incremental cost per QALY gained: ICER = [Cost (toripalimab plus chemotherapy) − Cost (placebo plus chemotherapy)]/[QALY (toripalimab plus chemotherapy) − QALY (placebo plus chemotherapy)].

The willingness-to-pay (WTP) threshold was defined as three times the *per capita* gross domestic product (GDP) of China in 2023, corresponding to $38,042.49 and $150,000.00 for the United States(Ding et al., 2025; Dong et al., 2025), following the WHO recommendations (Murray et al., 2000; Neumann et al., 2014). The analysis also included the incremental net health benefit (INHB) and incremental net monetary benefit (INMB), calculated as follows: INHB (λ) = (μE1 - μE0) - (μC1 - μC0)/λ = ΔE - ΔC/λ and INMB (λ) = (μE1 − μE0) × λ − (μC1 − μC0) = ΔE × λ − ΔC, where μCi and μEi are the costs and utility values associated with the toripalimab plus chemotherapy regimens (i = 1) or placebo plus chemotherapy (i = 0) regimens, and λ represents the WTP threshold.

### Clinical data input

A previously published method was used to construct survival curves for OS and PFS in the RATIONALE-312 trial (Guyot et al., 2012). The GetData Graph Digitizer (version 2.26, www.getdata.graph.digitizer.com) was used to digitize time-to-event data from the Kaplan-Meier survival curves for OS and PFS. Various parametric survival models including, Exponential, Weibull, Weibull proportional hazards (Weibull PH), Gamma, Log-normal, Gompertz, Generalized Gamma, and Log-logistic distributions, were used to extract data points.

**FIGURE 2:**
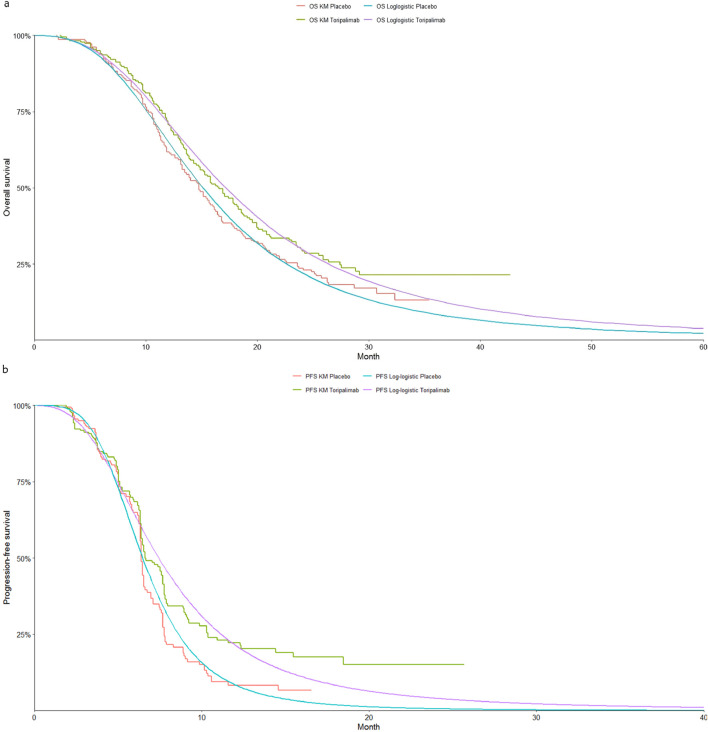
The Kaplan-Meier overall survival curves. **(a)** Overall survival, **(b)** Progression-free survival.

**FIGURE 3:**
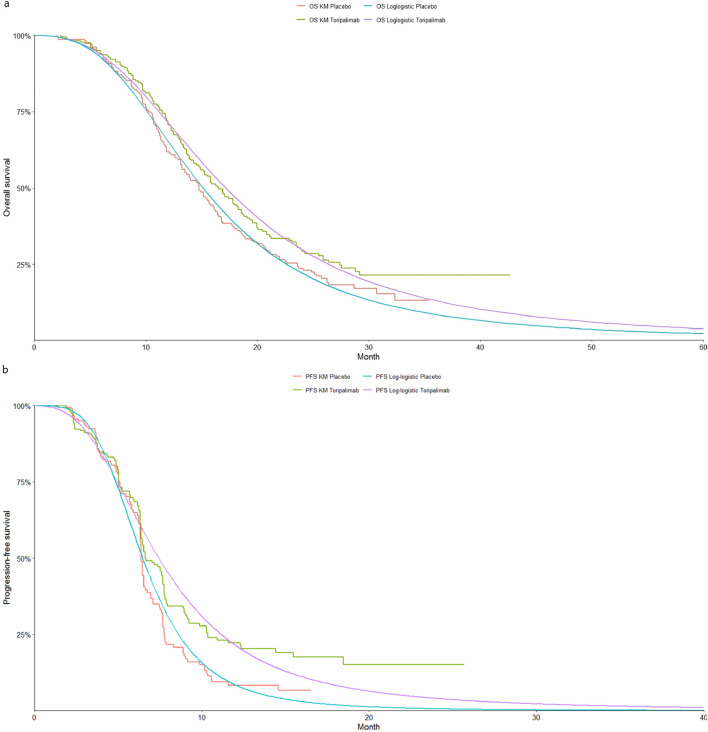
The Kaplan-Meier overall survival curves for patients with ITH-L. **(a)** Overall survival, **(b)** Progression-free survival.

**FIGURE 4:**
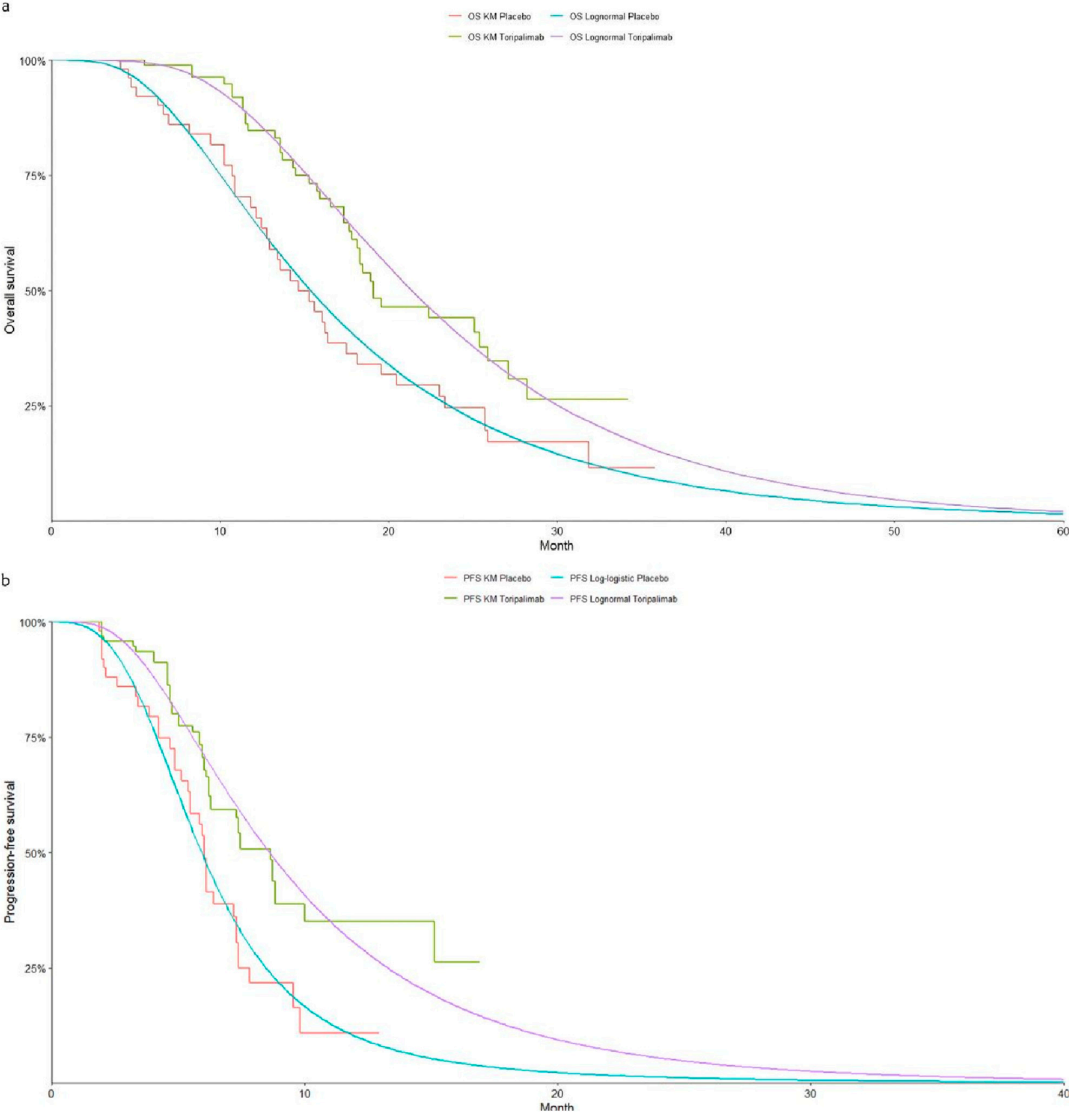
The Kaplan-Meier overall survival curves for patients with HLA-A11+/B62-. **(a)** Overall survival, **(b)** Progression-free survival.

The selection of the most suitable survival curves for PFS and OS was guided by assessments using the Akaike Information Criterion (AIC) and Bayesian Information Criterion (BIC), supported by visual inspection of the fitted curves. Table 1 provides each model’s estimated shape (g) and scale (*λ*) parameters. Further details on long-term survival data are presented in Tables 4–6 and Figures 2–4.

**TABLE 4:** The Akaike information criteria (AIC) and Bayesian information criteria (BIC).

Type of distribution	Toripalimab plus chemotherapy (OS)		Placebo plus chemotherapy (OS)		Toripalimab plus chemotherapy (PFS)		Placebo plus chemotherapy (PFS)	
	AIC	BIC	AIC	BIC	AIC	BIC	AIC	BIC
Exponential	1,240.0290	1,243.4360	1,260.0970	1,263.4860	864.9728	868.3800	768.0160	771.4050
Gamma	1,171.6110	1,178.4260	1,176.8070	1,183.5850	799.8772	806.6916	635.2843	642.0625
Generalized gamma	1,164.2260	1,174.4470	1,172.4790	1,182.6470	776.9676	787.1891	633.3803	643.5475
Gompertz	1,215.7410	1,222.5550	1,222.4340	1,229.2120	854.2128	861.0272	701.2803	708.0585
Weibull	1,183.4780	1,190.2920	1,188.9210	1,195.6990	817.4874	824.3017	653.3102	660.0883
WeibullPH'	1,161.2000	1,168.0140	1,168.0280	1,174.8060	817.4874	824.3017	653.3102	660.0883
Log-logistic	1,161.2000	1,168.0140	1,168.0280	1,174.8060	780.0376	786.8519	625.0739	631.8521
Lognormal	1,162.5260	1,169.3400	1,170.5080	1,177.2860	781.2917	788.1060	631.4413	638.2195

OS, overall survival; PFS, progression-free survival; AIC, akaike information criterion; BIC, bayesian information criterion.

**TABLE 5:** The Akaike information criteria (AIC) and Bayesian information criteria (BIC) of patients with ITH-L.

Type of distribution	Toripalimab plus chemotherapy (OS)		Placebo plus chemotherapy (OS)		Toripalimab plus chemotherapy (PFS)		Placebo plus chemotherapy (PFS)	
	AIC	BIC	AIC	BIC	AIC	BIC	AIC	BIC
Exponential	330.5549	332.7744	467.5742	470.0169	268.6060	270.8255	351.1980	353.6406
Gamma	325.8386	330.2776	440.7276	445.6129	262.0757	266.5147	298.7040	303.5893
Generalized gamma	326.6067	333.2652	431.5464	438.8744	258.7887	265.4472	299.1890	306.5169
Gompertz	331.1119	335.5509	459.7345	464.6198	269.9976	274.4366	323.4500	328.3353
Weibull	327.0066	331.4456	446.6638	451.5492	264.5628	269.0019	304.8615	309.7468
WeibullPH'	327.0066	331.4456	446.6638	451.5492	264.5628	269.0019	304.8615	309.7468
Log-logistic	323.8783	328.3173	436.2306	441.1159	257.7820	262.2210	297.0506	301.9359
Lognormal	324.6991	329.1382	434.0331	438.9184	257.5093	261.9483	297.1891	302.0744

OS, overall survival; PFS, progression-free survival; AIC, akaike information criterion; BIC, bayesian information criterion; ITH-L, low intratumor heterogeneity.

**TABLE 6:** The Akaike information criteria (AIC) and Bayesian information criteria (BIC) of patients with HLA-A11+/B62-.

Type of distribution	Toripalimab plus chemotherapy (OS)		Placebo plus chemotherapy (OS)		Toripalimab plus chemotherapy (PFS)		Placebo plus chemotherapy (PFS)	
	AIC	BIC	AIC	BIC	AIC	BIC	AIC	BIC
Exponential	364.1528	366.7875	308.7186	310.6699	326.4818	329.1165	218.1105	220.0618
Gamma	321.1152	326.3846	289.8667	293.7692	297.7616	303.0310	193.6630	197.5654
Generalized gamma	319.8627	327.7669	289.6635	295.5172	295.7284	303.6325	195.5209	201.3747
Gompertz	338.3224	343.5919	300.9050	304.8075	315.9746	321.2440	203.0393	206.9417
Weibull	325.9785	331.2479	292.8411	296.7436	302.6443	307.9138	195.3103	199.2128
WeibullPH'	325.9785	331.2479	292.8411	296.7436	302.6443	307.9138	195.3103	199.2128
Log-logistic	320.1675	325.4370	288.1730	292.0755	294.8727	300.1422	193.5773	197.4798
Lognormal	318.6670	323.9364	287.7892	291.6917	294.2969	299.5664	193.8541	197.7566

OS, overall survival; PFS, progression-free survival; AIC, akaike information criterion; BIC, bayesian information criterion.

#### Cost input

Only direct medical costs were analyzed, including drug expenses, laboratory test fees, PET-CT scans, prophylactic medications for intravenous treatments, best supportive care, end-of-life care, drug administration, subsequent treatments, and management of SAEs. To determine the costs of medications, local charges from the China Health Industry Data Platform (https://data.yaozh.com/) were utilized (Yaoch, 2025), using the national median price as the reference point, while other cost-related data were obtained from previous studies and relevant publications.

Drug doses followed the RATIONALE-312 study protocol, and treatment cycle costs were calculated accordingly using local price data (Tables 1–3) (Wong et al., 2018; Liu et al., 2021; Cao et al., 2022; Zhu et al., 2022; Liu et al., 2023a; Shao et al., 2023; Centers_for_Medicare_and_Medicaid_Services, 2025; Yaoch, 2025). The AE-related costs were determined by multiplying the estimated incidence rates by the respective treatment expenses. All AEs were assumed to occur during the initial treatment cycle; detailed incidence rates are provided in Tables 1–3 (Wong et al., 2018; Zhu et al., 2021; Jiang and Wang, 2022; Zhu et al., 2022; Liu et al., 2023b; Shao et al., 2023).

#### Utility inputs

Health utility scores range from 0 (death) to 1 (perfect health). As the RATIONALE-312 trial did not report quality of life outcomes, utility values were obtained from published literature. The utility scores for PFS and PD were assigned as 0.69 and 0.60 (Vedadi et al., 2021) (Table 2 **)**. The impact of AEs on health utility (disutility) was considered only during the first cycle of the model (Zhu et al., 2021; Zhu et al., 2022; Liu et al., 2023b).

#### Sensitivity analysis

One-way sensitivity analysis (OWSA) and probabilistic sensitivity analysis (PSA) were performed to address the model uncertainty. In the OWSA, the literature informed parameter ranges, with values fluctuating by ±20% from the baseline estimates. For PSA, the model parameters were varied simultaneously in 10,000 Monte Carlo simulations to estimate the likelihood of cost-effectiveness for each intervention at different WTP thresholds per additional QALY. Beta distributions were applied to the utility parameters, whereas gamma distributions were used for the cost variables. The results are presented as a scatter plot and cost-effectiveness acceptability curve.

#### Subgroup analyses

Subgroup analyses examined the cost-effectiveness of toripalimab plus chemotherapy versus chemotherapy alone as a first-line treatment for ES-SCLC in China and the United States These analyses focused on patients with ITH-L and A11+/B62−. As specific data on follow-up treatments, drug use, and AE incidence in these subgroups were unavailable from the EXTENTORCH trial, these characteristics were assumed to be aligned with those of the overall study population.

## Results

### Base-case analysis

Over a 10-year analysis horizon, the base-case results revealed that the toripalimab plus chemotherapy group achieved an additional 0.81 QALYs at an incremental cost of $16,515.81. In contrast, the chemotherapy-only group attained 0.71 QALYs for $11,827.35. The comparative analysis demonstrated an average incremental effect of 0.10 QALYs at an added cost of $4,688.46 for the toripalimab regimen, resulting in an ICER of $45,629.27 per QALY for toripalimab plus chemotherapy compared to chemotherapy alone (Table 7).

**TABLE 7:** The base case analysis.

Treatment	Cost	QALY	Incremental cost	Incremental QALY	INHB	INMB	ICER
Toripalimab plus chemotherapy (China)	16,515.81	0.81	4,688.46	0.10	−0.02	−779.55	45,629.27
Chemotherapy (China)	11,827.35	0.71					
Toripalimab plus chemotherapy (China) (ITH-L)	21,306.20	1.18	7,534.87	0.34	0.14	5,292.72	22,345.99
Chemotherapy (China) (ITH-L)	13,771.32	0.84					
Toripalimab plus chemotherapy (China) (A11+/B62–)	17,968.93	0.90	6,122.63	0.20	0.04	1,423.20	30,867.38
Chemotherapy (China) (A11+/B62–)	11,846.30	0.70					
Toripalimab plus chemotherapy (United States)	167,278.59	0.83	90,483.17	0.11	−0.50	−74380.20	842,855.23
Chemotherapy (United States)	76,795.42	0.72					
Toripalimab plus chemotherapy (ITH-L) (United States)	200,248.24	1.22	117,073.22	0.36	−0.42	−63646.78	328,694.61
Chemotherapy (ITH-L) (United States)	83,175.02	0.86					
Toripalimab plus chemotherapy (A11+/B62–) (United States)	183,732.16	0.92	105,797.65	0.20	−0.50	−75303.26	520,412.03
Chemotherapy (A11+/B62–) (United States)	77,934.51	0.71					

QALY, Quality-adjusted life year, ICER, Incremental cost-effectiveness ratio, INMB:the incremental net monetary benefits, INHB, the incremental net health benefits, ITH-L, low intratumor heterogeneity.

When assessed against China’s WTP threshold of $38,042.49 per QALY, toripalimab plus chemotherapy was not cost-effective compared with chemotherapy alone. INHB was calculated as −0.02 QALYs, with an INMB of $-779.55 (Table 7). The ICER for toripalimab plus chemotherapy in the United States was $842,855.23 per QALY, far exceeding the United States WTP threshold of $150,000.00 per QALY. At this threshold, the INHB was −0.50 QALYs, and the INMB was $-74,380.20 compared to chemotherapy alone (Table 7).

### Subgroup analysis

In subgroup analyses, the ICER for toripalimab plus chemotherapy compared to chemotherapy alone was $22,345.99 per QALY gained for patients with ITH-L and $30,867.38 per QALY for those with A11+/B62-, both falling below China’s WTP threshold of $38,042.49 per QALY (Table 7). The INHB for toripalimab plus chemotherapy was calculated as 0.14 QALYs for ITH-L patients and 0.04 QALYs for A11+/B62- patients. The corresponding INMB values were $5,292.72 and $1,423.20, respectively, at the WTP threshold of $38,042.49 per QALY (Table 7).

In contrast, in the United States, the ICER for toripalimab plus chemotherapy compared to chemotherapy alone was $328,694.61 per QALY for ITH-L patients and $520,412.03 per QALY for A11+/B62- patients, exceeding the United States WTP threshold of $150,000.00 per QALY (Table 7). The INHB for toripalimab plus chemotherapy was −0.42 QALYs for ITH-L patients and −0.50 QALYs for A11+/B62- patients, while INMB values were $-63,646.78 and $-75,303.26, respectively, at a WTP threshold of $150,000.00 per QALY (Table 7).

### Sensitivity analysis

**FIGURE 5:**
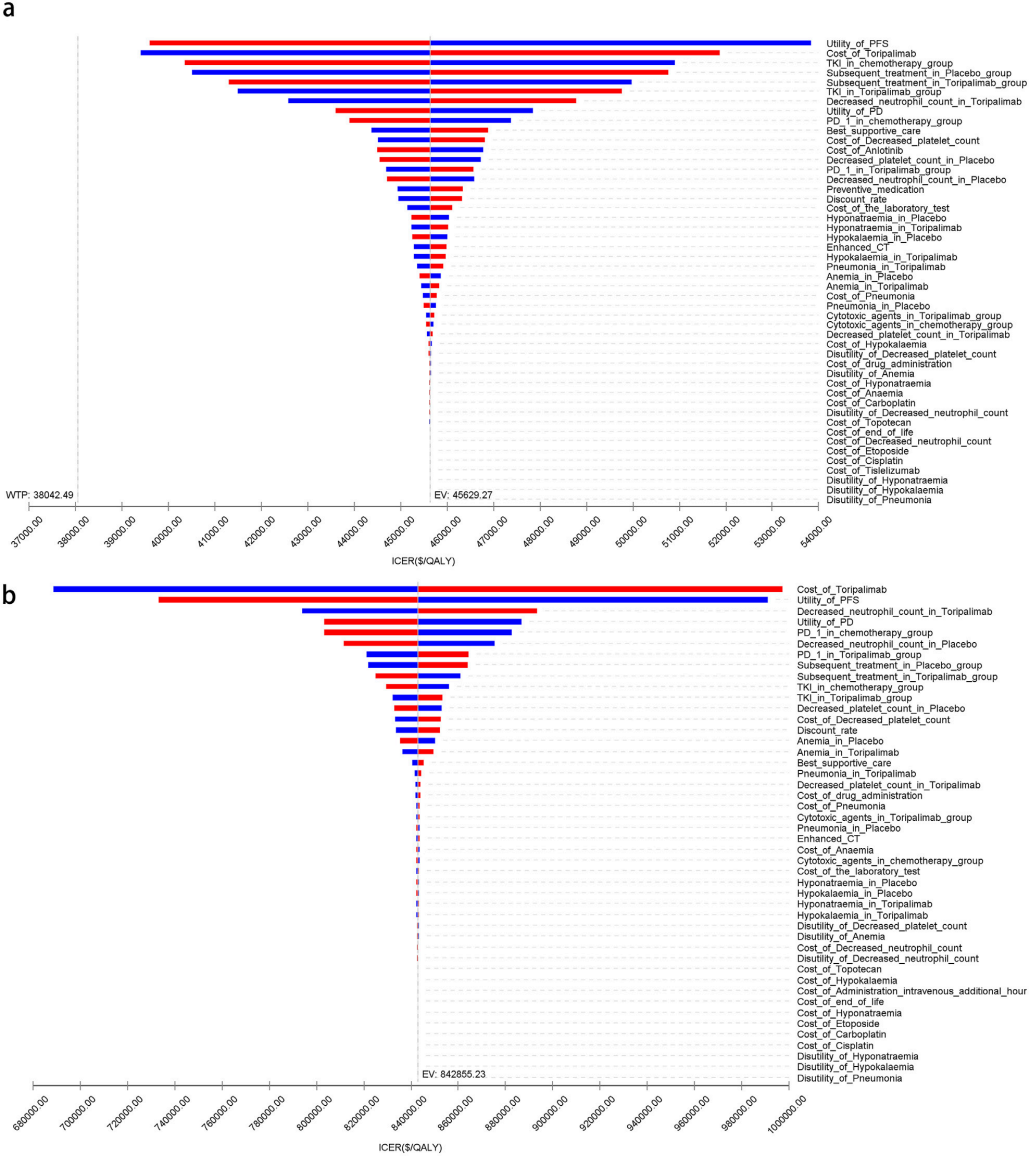
The tornado diagram of one-way sensitivity analysis. **(a)** China, **(b)** The United States.

**FIGURE 6:**
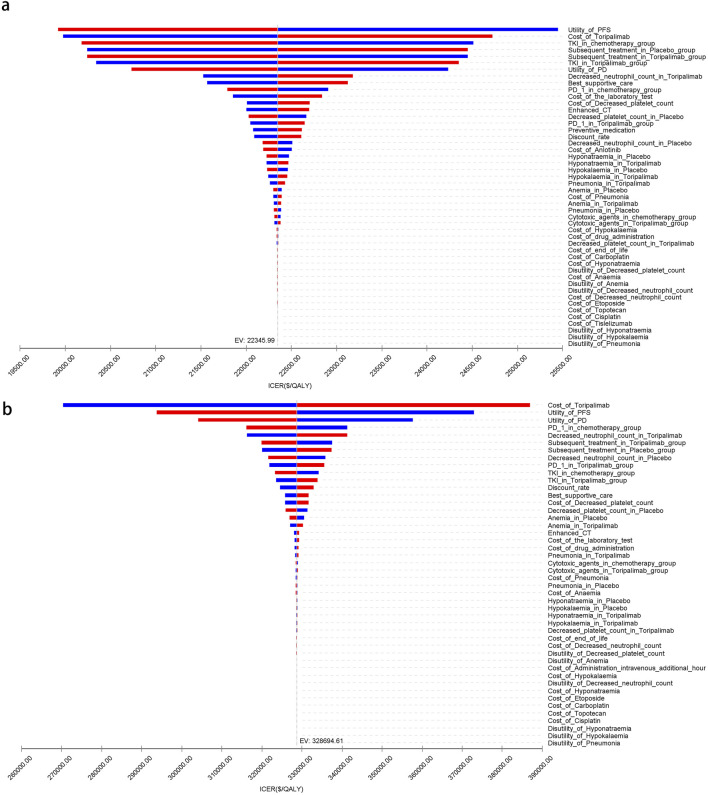
The tornado diagram of one-way sensitivity analysis for patients with ITH-L. **(a)** China, **(b)** The United States.

**FIGURE 7:**
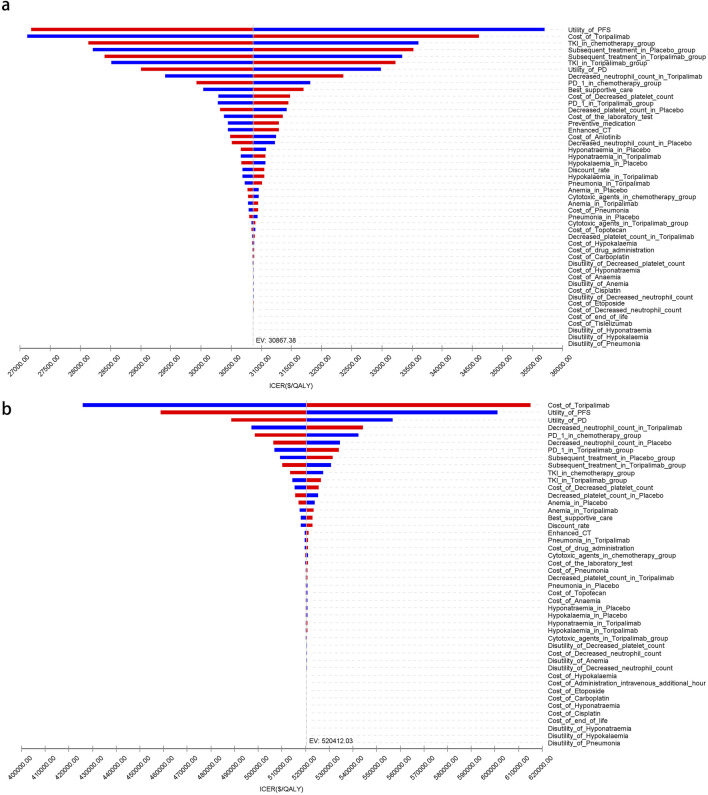
The tornado diagram of one-way sensitivity analysis for patients with A11+/B62-. **(a)** China, **(b)** The United States.

Figures 5–7 present a tornado diagram from the OWSA, analyzing China’s entire population and subgroups. It highlights the factors most significantly affecting base-case outcomes, including the utility value of PFS, the cost of toripalimab, and the proportion of tyrosine kinase inhibitors used in subsequent treatments in the toripalimab plus chemotherapy group. Figures 5–7 show that, for United States patients, the ICER was most influenced by the cost of toripalimab, the utility value of PFS, and the utility value of PD. However, due to substantial differences in health outcomes between the two treatment strategies in China and the United States, the parameter values did not alter the overall study conclusions.

**FIGURE 8:**
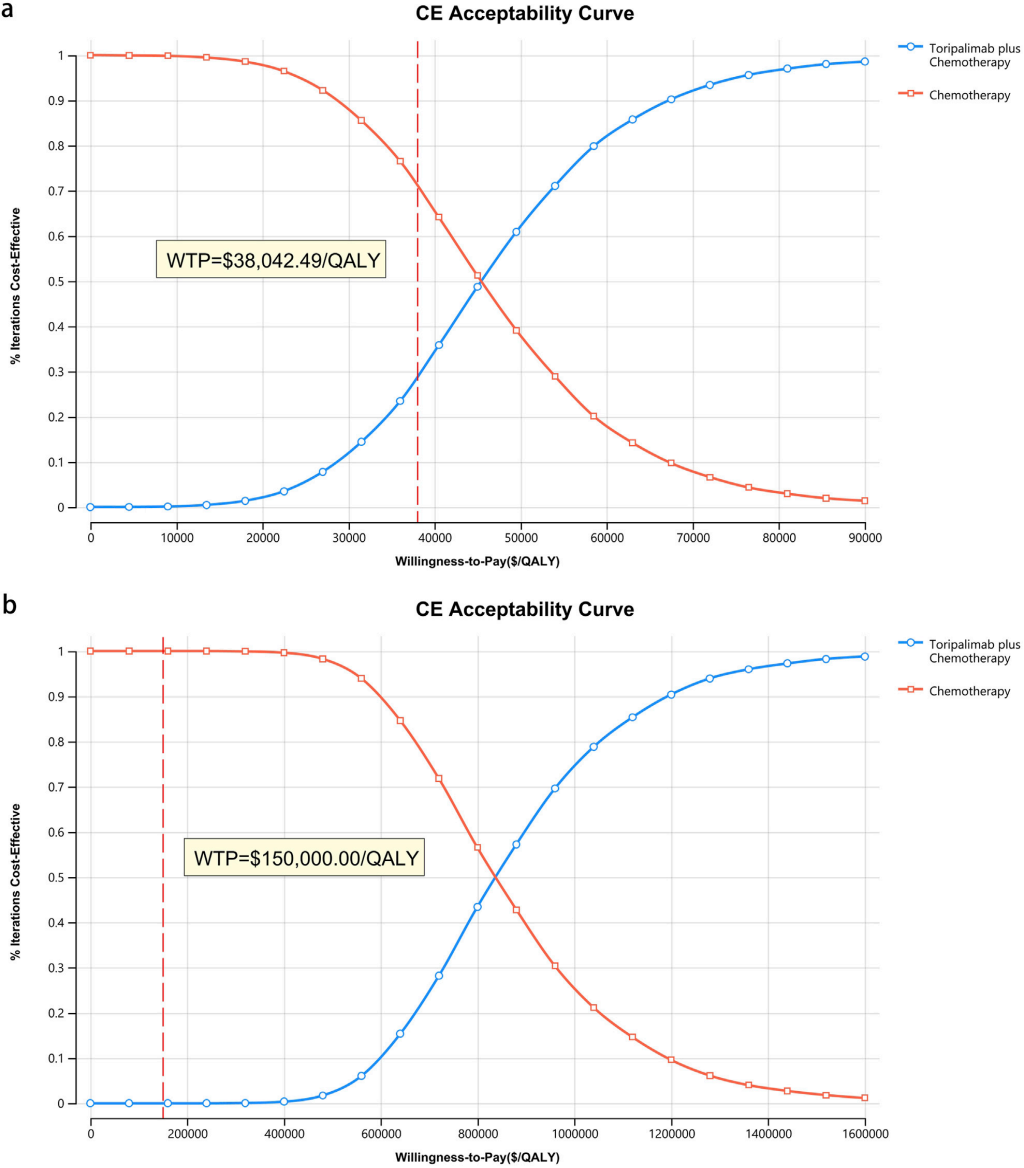
The cost-effectiveness acceptability curve. **(a)** China, **(b)** The United States.

**FIGURE 9:**
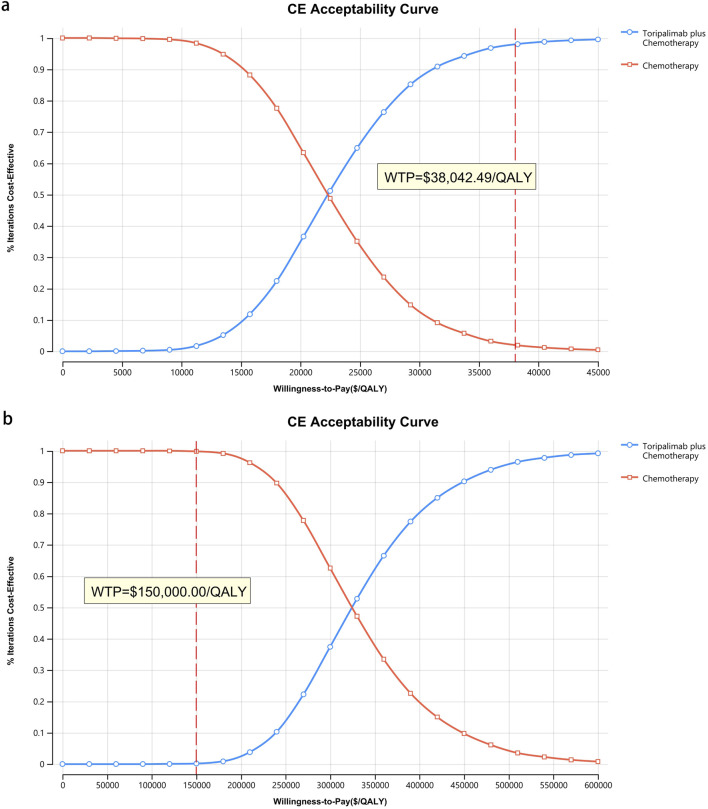
The cost-effectiveness acceptability curve for patients with ITH-L. **(a)** China, **(b)** The United States.

**FIGURE 10:**
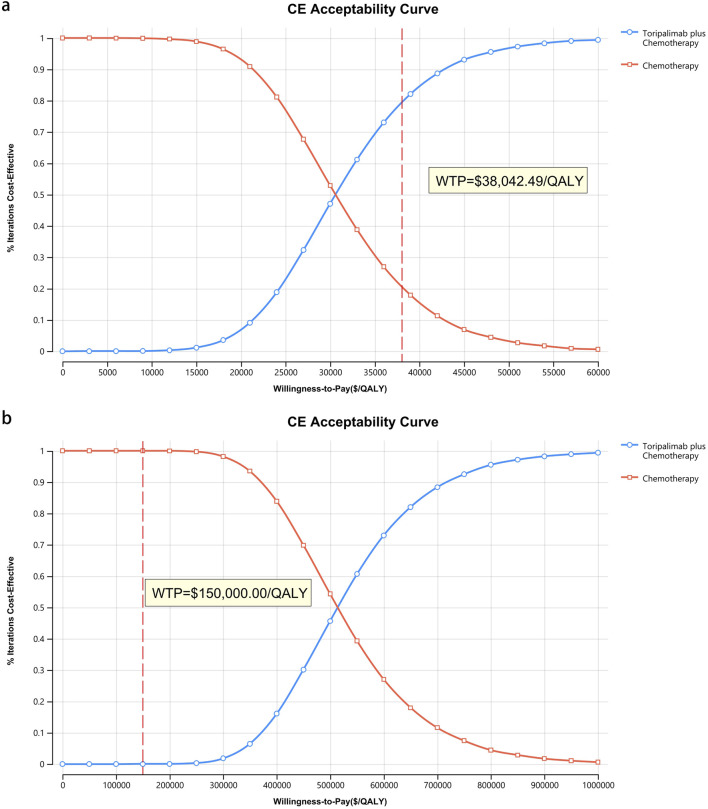
The cost-effectiveness acceptability curve for patients with A11+/B62-. **(a)** China, **(b)** The United States.

Figures 8–10 provide acceptability curves and probabilistic scatter plots, clearly representing the cost-effectiveness landscape. These tools, essential for decision-making, illustrate the probability that toripalimab plus chemotherapy is cost-effective at different WTP thresholds, and as the WTP increases, the likelihood of cost-effectiveness in the toripalimab plus chemotherapy group grows. At a WTP threshold of $150,000.00 in the United States, the acceptability curves revealed that almost 0% probability of serplulimab plus chemotherapy was cost-effective in various groups (the overall population, ITH-L subgroup, and A11+/B62- subgroup).

**FIGURE 11:**
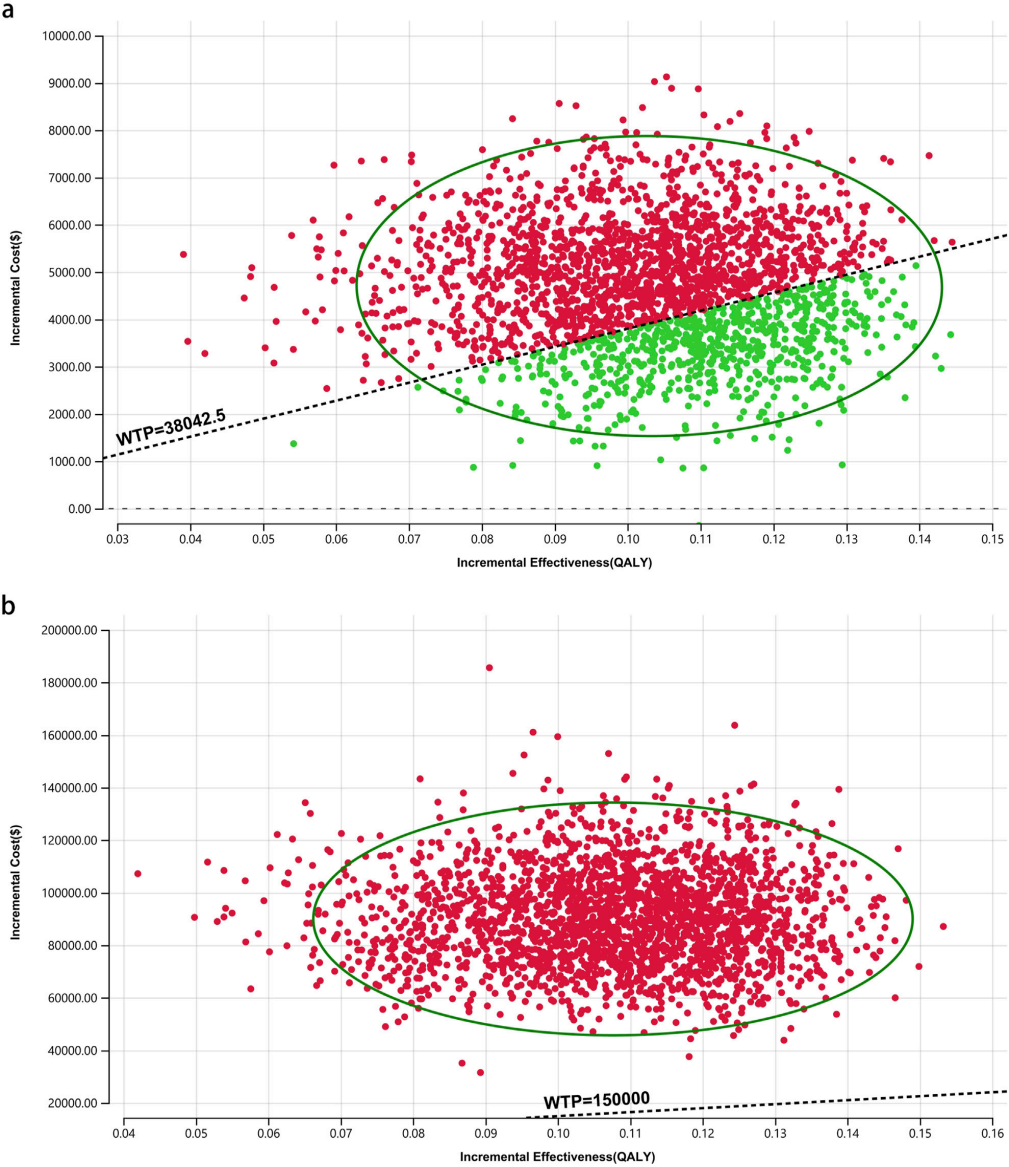
The cost-effectiveness probabilistic scatter plot. **(a)** China, **(b)** The United States.

**FIGURE 12:**
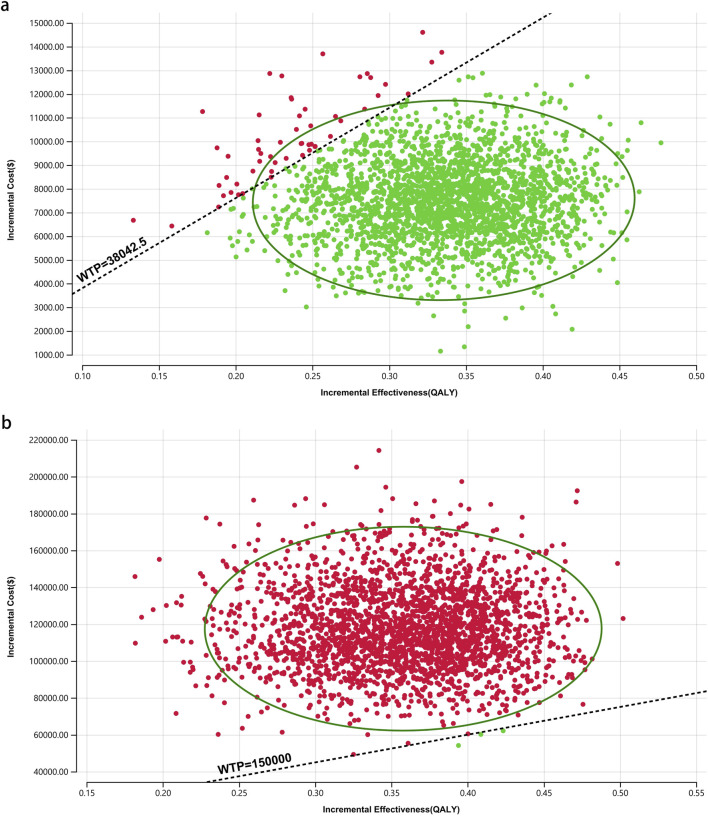
The cost-effectiveness probabilistic scatter plot for patients with ITH-L. **(a)** China, **(b)** The United States.

**FIGURE 13:**
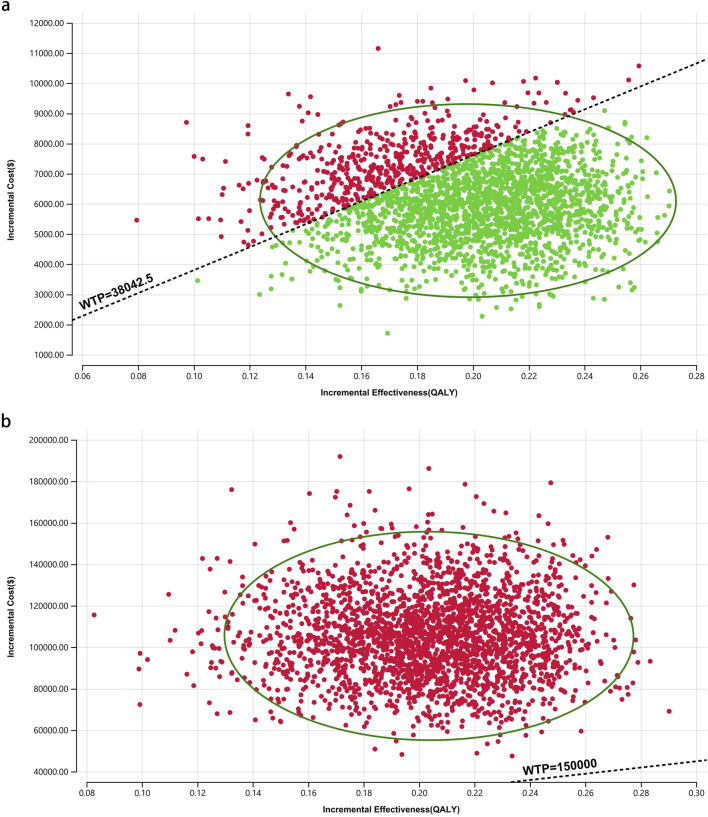
The cost-effectiveness probabilistic scatter plot for patients with A11+/B62-. **(a)** China, **(b)** The United States.

In China, these probabilities were 29.95% for the overall population, 97.88% for patients with ITH-L, and 79.50% for the A11+/B62- subgroup, evaluated against a WTP threshold ($38,042.49) (Figures 11–13). In the United States, the probabilities were 0% for the entire population, 0.16% for ITH-L patients, and 0% for the A11+/B62- subgroup, based on a WTP threshold of $150,000.00 (Figures 11–13).

## Discussion

This study represents the first evaluation of the cost-effectiveness of toripalimab combined with chemotherapy as first-line therapy for ES-SCLC. Our findings indicate that this combination is not a cost-effective option from the perspective of the Chinese healthcare system or the United States payer. This conclusion is supported by the INHB and INMB results, which were −0.02 and −0.50 QALYs and $-779.55 and $-74,380.20 for China and the United States, respectively.

Previous economic evaluations of ES-SCLC treatments in China and the United States (Zhou et al., 2019; Ding et al., 2021; Liu et al., 2021; Wang et al., 2021; Zhu et al., 2021; Zhu et al., 2022; Gan et al., 2023; Long et al., 2023; Shao et al., 2023; Xiang et al., 2023) have similarly highlighted the significant financial burden imposed by ICIs, despite notable gains in QALYs. Many studies have suggested that ICIs may not provide cost-effective alternatives to chemotherapy, with drug costs for PD-1/PD-L1 antibodies being a significant determinant of outcomes in both countries.

Our findings provide growing evidence supporting the use of cost-effective, domestically manufactured anticancer therapies. They highlight the implications for the Chinese government, as it seeks to balance finite healthcare resources with an increasing demand for cancer treatments. Clinically, we recommend that physicians tailor treatment plans based on the patient’s disease status and financial capabilities, prioritizing affordable and effective therapies. In the United States, the ICER for toripalimab plus chemotherapy significantly exceeded the WTP threshold of $150,000.00 per QALY, with a cost-effectiveness acceptability curve showing a 0% probability that the combination is cost-effective.

For SCLC, racial disparities in disease characteristics—associated with differential gene expression and transcriptional subtypes—lead to distinct therapeutic responses and prognoses (Liu et al., 2023c). Asians generally exhibit lower treatment tolerance and different economic baselines than Caucasians and Africans, potentially influencing treatment access and survival outcomes. In the United States, CMS Part B reimbursement schedules reflect clinician-administered drug payments rather than actual acquisition costs (Centers_for_Medicare_and_Medicaid_Services, 2025), which may overestimate drug expenses and bias cost-effectiveness results. However, given the ICER’s substantial exceedance of the WTP threshold, this bias does not alter the study’s conclusions.

Our findings add to the growing evidence supporting the adoption of cost-effective, domestically manufactured anticancer therapies. These insights underscore critical implications for health policymakers, who must balance finite healthcare resources against the escalating demand for cancer treatments. Clinically, we recommend that physicians customize treatment plans based on patients’ disease profiles and financial capacities, prioritizing affordable yet efficacious therapies. This evidence base can inform adjustments to health insurance reimbursement directories by medical insurance bureaus and guide the recommended tiering of drugs in clinical practice guidelines.

In the United States, the ICER for toripalimab plus chemotherapy substantially exceeded the $150,000.00 per QALY WTP threshold, with cost-effectiveness acceptability curves showing a 0% probability of cost-effectiveness. OWSA identified toripalimab costs and utility values as the most influential drivers of ICER in both China and the United States To address this, patient assistance programs could be leveraged to support low-income patients, particularly those with PD-1 progression, intolerance to ICIs, or limited access to alternative therapies. Alternatively, price reductions for toripalimab could enhance accessibility and benefit a broader patient population.

Similar to other analyses, our research confirms that the combination of ICIs and chemotherapy does not achieve cost-effectiveness for ES-SCLC due to the high cost of the drugs and limited improvements in efficacy. There are some differences in the economic analysis standards adopted by different institutions. Therefore, this study highlights the need for more evaluations, including network meta-analyses (NMA) and cost-effectiveness studies that incorporate the perspectives of United States payers, to comprehensively compare ICIs.

Toripalimab will be marketed globally to benefit all patients. Given that only economic analyses of the United States and China were conducted, developed countries such as Europe, America, Japan, and South Korea can refer to the economic analysis of the United States, whereas developing countries in Asia, Africa, and Latin America can refer to the economic analysis results of China.

The EXTENTORCH trial demonstrated that the therapeutic effects on PFS and OS were independent of tumor PD-L1 expression or TMB status, consistent with findings from other phase 3 studies in ES-SCLC. This finding highlights the need for novel biomarkers. ITH has emerged as a potential predictive biomarker of SCLC treatment outcomes associated with improved OS and PFS (Chowell et al., 2018). Additionally, HLA-I genes affect the survival of patients with ICIs. For example, the HLA-B44 supertype is associated with prolonged survival, whereas HLA-B62 or somatic loss of heterozygosity in HLA-I is associated with worse outcomes (Chowell et al., 2018). Biomarker analysis in EXTENTORCH revealed that patients with a lower ITH or HLA-A11+ HLA-B62- haplotype showed more favorable responses to toripalimab plus chemotherapy (Stewart et al., 2020; George et al., 2024).

Given that the HLA-A11 haplotype is more prevalent in East Asians (approximately 30%) than in white individuals (5%–10%), further research is needed to clarify the roles of HLA-A11 and B62 in presenting neoantigens from SCLC tumors. Unlike other studies that do not have subgroup analysis, our subgroup analysis uniquely showed that patients with ITH-L and HLA-A11+ HLA-B62 had lower ICERs than the overall population in China and the United States These subgroups present a cost-effective first-line treatment option for ES-SCLC in China, offering potentially more favorable patient alternatives.

Sensitivity analysis identified the utility values of PFS and PD as critical determinants of cost-effectiveness outcomes. Owing to the lack of EQ-5D and cost-per-QALY data in the EXTENTORCH trial, utility values were derived from the literature, introducing some uncertainty. However, unlike many studies that rely on utility data from non-small cell lung cancer (NSCLC), our study used data specific to SCLC, thus improving the accuracy and relevance of the results.

This study has several limitations. First, clinical data were derived from a phase 3 trial conducted in China, whereas the cost-effectiveness evaluation incorporated a United States perspective, potentially introducing bias. Second, management costs and disutility associated with grade 1–2 AEs were excluded. Although the toripalimab plus chemotherapy group exhibited a higher incidence of these AEs, which could theoretically increase costs and reduce QALYs, the absolute difference in AE rates between groups was minimal. One-way sensitivity analysis further indicated that grade 1–2 AEs had a low impact on ICER, suggesting limited influence on overall results. Additionally, while the longer survival period in the toripalimab group might incur higher indirect costs (e.g., supportive care), sensitivity analysis showed that variations in best supportive care costs did not affect outcomes, implying minimal impact of indirect costs on conclusions. Third, CMS Part B physician fee schedules reflect reimbursement for services and clinician-administered drugs, representing provider payments rather than actual drug acquisition costs. The lack of adjustment for rebates may overestimate drug expenses, potentially biasing cost-effectiveness results against the treatment. In addition, for the general population, the toripalimab plus chemotherapy group only needs 6 years to achieve 99% mortality rate, to be consistent with the subgroup used it for 10 years, this will overestimate ICER. However, it has little impact on the results. Despite these challenges, this cost-effectiveness analysis based on EXTENTORCH data provides critical insights for treatment decision making.

## Conclusion

This investigation revealed that toripalimab combined with chemotherapy is a cost-effective first-line treatment for patients with ES-SCLC with ITH-L and A11 +/B62-histology in China. However, this combination is not cost-effective for the overall patient population in China or any patient group and subgroup in the United States These findings provide critical information for policymakers and healthcare professionals and offer evidence to support the broader application of toripalimab in clinical practice worldwide.

## Data Availability

The original contributions presented in the study are included in the article/Supplementary Material, further inquiries can be directed to the corresponding author.
